# Fas-deficient mice have impaired alveolar neutrophil recruitment and decreased expression of anti-KC autoantibody:KC complexes in a model of acute lung injury

**DOI:** 10.1186/1465-9921-13-91

**Published:** 2012-10-09

**Authors:** Sucheol Gil, Alex W Farnand, William A Altemeier, Sean E Gill, Anna Kurdowska, Agnieszka Krupa, Jon M Florence, Gustavo Matute-Bello

**Affiliations:** 1 The Center for Lung Biology, Division of Pulmonary & Critical Care Medicine, Department of Medicine, University of Washington, 850 Republican Street, Box 351082, Seattle, WA, 98109, USA; 2 The Centre for Critical Illness Research, Lawson Health Research Institute, Department of Medicine, Western University, London, ON, Canada; 3 The Department of Cellular and Molecular Biology, University of Texas Health Science Center, Tyler, TX, USA; 4 The Medical Research Service, Seattle VA Puget Sound Healthcare System, Seattle, WA, USA

**Keywords:** Inflammation, Fas, Cytokines, Mechanical ventilation, Apoptosis, Lipopolysaccharide

## Abstract

**Background:**

Exposure to mechanical ventilation enhances lung injury in response to various stimuli, such as bacterial endotoxin (LPS). The Fas/FasL system is a receptor ligand system that has dual pro-apoptotic and pro-inflammatory functions and has been implicated in the pathogenesis of lung injury. In this study we test the hypothesis that a functioning Fas/FasL system is required for the development of lung injury in mechanically ventilated mice.

**Methods:**

C57BL/6 (B6) and Fas-deficient *lpr* mice were exposed to either intra-tracheal PBS followed by spontaneous breathing or intra-tracheal LPS followed by four hours mechanical ventilation with tidal volumes of 10 mL/kg, respiratory rate of 150 breaths per minute, inspired oxygen 0.21 and positive end expiratory pressure (PEEP) of 3 cm of water.

**Results:**

Compared with the B6 mice, the *lpr* mice showed attenuation of the neutrophilic response as measured by decreased numbers of BAL neutrophils and lung myeloperoxidase activity. Interestingly, the B6 and *lpr* mice had similar concentrations of pro-inflammatory cytokines, including CXCL1 (KC), and similar measurements of permeability and apoptosis. However, the B6 mice showed greater deposition of anti-KC:KC immune complexes in the lungs, as compared with the *lpr* mice.

**Conclusions:**

We conclude that a functioning Fas/FasL system is required for full neutrophilic response to LPS in mechanically ventilated mice.

## Background

Acute lung injury (ALI) and its more severe form, the acute respiratory distress syndrome (ARDS) remain important clinical problems in the United States, with an incidence rate of 38.3 cases per 100,000 person-years and a mortality rate of 45% [1]. ALI/ARDS is characterized clinically by sudden respiratory failure with impaired oxygenation and non-cardiogenic pulmonary edema [2]. Pathologically ALI/ARDS is associated with an early inflammatory phase with neutrophilic alveolitis and destruction of the alveolar/capillary permeability barrier, followed by a late fibroproliferative phase with abnormal repair and collagen deposition. There are no specific treatments for ARDS and the main supportive treatment, mechanical ventilation, can be harmful to the lungs when delivered at high tidal volumes [3].

A growing body of experimental evidence suggests that in addition to the injury caused by high tidal volumes, even moderate or low tidal volumes markedly enhance injury when the lungs are exposed to pathogens or their products [4-6]. For example, mechanical ventilation synergistically enhances lung injury in response to low doses of bacterial lipopolysaccharide (LPS) and this is associated with expression of specific sets of genes that aren’t expressed with LPS or ventilation alone [4,5,7]. A computational analysis has mapped the biological processes that are activated by the combination of mechanical ventilation and LPS [8]. One of these biological processes is apoptosis, and a known mediator of apoptosis in injured lungs is the Fas/FasL system.

The Fas/FasL system is composed of the membrane surface receptor Fas and its cognate ligand, FasL. FasL exists in a membrane-bound form, and also in a soluble form that is present in the lungs during acute lung injury [9]. Binding of Fas to sFasL in alveolar epithelial cells independently activates apoptotic and inflammatory pathways that result in death of the cells but also in release of pro-inflammatory cytokines [10]. Although the Fas/FasL system is better known for its pro-apoptotic function, the pro-inflammatory function is also important in the development of lung injury, and mice deficient in Fas (*lpr*) have an impaired neutrophil recruitment in response to LPS installation and bacterial infections [11].

Several lines of evidence suggest that the Fas/FasL system plays a role in ALI/ARDS. First, bioactive sFasL is present in the lungs of patients with ARDS, and this is associated with increased mortality [9,12]. Second, genetic variations in the Fas gene are associated with increased risk for ALI/ARDS in humans [13]. Third, activation of the Fas/FasL system in mammals leads to acute inflammatory injury followed by fibrosis [14-17]. And finally, mice lacking Fas are protected in a number of models of lung injury [11,18-20]. Thus, the apoptotic and inflammatory responses induced by Fas activation in the lungs are one important factor in the development of ALI/ARDS.

One of the most important neutrophil chemoattractants in mice is the chemokine KC (CXCL1), which is the murine functional homologue of the human chemokine IL8 (CXCL8). Activation of Fas in alveolar epithelial cells is followed by a marked increase in KC expression [10]. Interestingly, recent studies suggest that in injured lungs, IL8 in humans and KC in mice form immune complexes with autoantibodies, and these immune complexes can in turn bind Fc receptors such as FcγRIIa and FcγRIII that amplify the inflammatory response [21,22]. These studies suggest that, in addition to the absolute levels of chemokines, the formation of autoimmune complexes and their association with Fc receptors is important for the development of inflammatory responses in the lungs.

In this study, we ask whether the Fas/FasL system plays a role in the amplification of inflammatory responses that occur early in the course of mechanical ventilation. The specific hypothesis to be tested is that the Fas/FasL system is required for the development of lung injury in mechanically ventilated mice exposed to LPS. To test this hypothesis, we investigate whether the combination of a non-injurious mechanical ventilation strategy with a minimal dose of intratracheal LPS results in an acute lung injury, and whether this injury is attenuated in Fas-deficient *lpr* mice. We further investigate whether the development of injury is associated with formation and deposition of anti-KC:KC immune complexes.

## Materials and methods

### Animal protocol

All of the animal experiments were approved by the institutional animal research committee of the University of Washington. Mice were housed in a pathogen-free environment according to University of Washington animal use guidelines. Male C57BL/6 mice (“B6”) and mice carrying a spontaneous mutation in the Fas gene that impairs Fas signaling (B6. MRL-*Fas*^*lpr*^/J, “*lpr*”) were obtained from the Jackson Laboratories and studied at 7-13 weeks of age. Briefly, the mice were anesthetized with 5% inhaled isoflurane and then intubated endotracheally with a 20-gauge angiocatheter. Placement of the catheter in the trachea was verified by visualizing the movement of a 100 μl bubble of water in response to respiratory efforts. After confirming intubation, the trachea was instilled with either *E. coli* LPS, 15 ng/kg or PBS. The instillate was suspended in 2.5% colloidal carbon to allow later confirmation of the extent and distribution of the instillation macro and microscopically. After the installations some of the mice were extubated, returned to their cages, and allowed free access to food and water; other mice where kept intubated and subjected to mechanical ventilation with the following settings: tidal volume (Tv) 10 ml/kg; respiratory rate (RR) 150 breaths/minute; fraction of inspired oxygen (FiO_2_) 0.21; and positive end-expiratory pressure (PEEP) of 3 cm H_2_O. The heart rate, airway pressures, rectal temperatures and EtCO_2_ were monitored continuously using a computerized monitoring system (Chart 4, AD Instruments, Colorado Springs, CO). The RR was adjusted to maintain the EtCO_2_ between 30 – 40 mmHg. The body temperature was maintained between 37 and 38°C with external heating. The mice were hydrated with a continuous intraperitoneal infusion of lactated ringer solution at 500 μl/hour. Muscle relaxation was attained with pancuronium bromide, 1 μg/g i.p followed by 0.5 μg/g i.p. every hour. After four hours of mechanical ventilation the mice were euthanized with 0.30 ml/kg i.p. of Beuthanasia-D (Schering-Plough Animal Health, Union, NJ). The thorax was rapidly opened and the mouse was exsanguinated by direct cardiac puncture. The left lung was removed and flash-frozen in liquid nitrogen. The right lung was lavaged with 0.6 mM EDTA in PBS; an aliquot of the bronchoalveolar lavage fluid (BALF) was removed for cell counts and differentials, the remaining fluid was spun at 1200 x *g*, and the supernatants stored at -80°C in individual aliquots. Following the BAL, the right lung was fixed in 4% buffered paraformaldehyde at an inflation pressure of 15 cm H_2_O for histological evaluation.

### Experimental design

We studied four groups of mice. Two groups consisted of B6 and *lpr* mice instilled with PBS and allowed to breathe spontaneously (“SB”) (n = 7 for B6 mice, 4 for *lpr*). The other two groups consisted of B6 and *lpr* mice instilled with LPS and exposed mechanical ventilation (MV + LPS) (n = 10 for B6 mice, 6 for *lpr*). The main experimental comparison was between the B6 and *lpr* mice instilled with LPS and exposed to ventilation.

### Measurements

Total cell counts in the BALF were performed with a hemacytometer. Differential counts were performed on cytospin preparations using the Diff-quick method (Fisher Scientific Company L.L.C., Kalamazoo, MI). BALF total protein was measured with the bicinchoninic acid method (BCA assay, Pierce, Rockford, IL). BALF IgM (Bethyl Laboratories, Montgomery, TX) and α-macroglobulin (Life Diagnostics, West Chester, PA) were measured with immunoassays. Lung homogenate TNF-α, KC, IL1β, and IL6 were measured using a multiplex fluorescent bead assay (R&D Systems, Minneapolis, MN).

As a measurement of the total content of PMN in the lungs we measured myeloperoxidase (MPO) activity in lung homogenates prepared in 50 mM K_2_HPO_4_, pH 6.0 with 5% CH_3_(CH_2_)_15_ N(Br)(CH_3_)_3_, 5 mM EDTA. Active caspase-3 and Poly ADP ribose polymerase (PARP) activity were measured in lung homogenates prepared on a 1:20 ratio of a lysis buffer (0.5% Triton-X-100, 150 mM NaCl, 15 mM Tris, 1 mM CaCl, and 1 mM MgCl, pH7.4). The lung homogenate was spun at 10,000 x *g* for 20 min at 4°C and the supernatant used for measurements of active caspase-3 and PARP using the CPP32/Caspase-3-Fluorometric Protease Assay Kit (BioVision, Mountain View, CA) and a PARP activity kit (Cell Signaling, Boston MA). Serum Creatinine, ALT and bilirubin were measured at a commercial laboratory. Anti-KC autoantibody:KC immune complexes were measured in BAL fluids using an ELISA assay according to a previously described protocol [21]. Briefly, 96-well microtiter plates were coated with antibody against KC (Peprotech). After blocking, the plates were incubated with BAL fluid samples obtained from mice. Then, the plates were washed and incubated with biotinylated horse antibody against mouse immunoglobulins (Vector Laboratories) followed by HRP-conjugated streptavidin and the substrate tetramethyl benzidine (Sigma).

#### Measurement of murine neutrophil chemotaxis *in vitro*

C57BL/6 and *lpr* mice were euthanized by exposure to CO_2_ followed by cervical dislocation. The femur and tibia of both hind legs were isolated and freed of all soft tissue, and then the ends of both bones were removed. The femur and tibia were placed proximal end down in a 0.6 mL Eppendorf tube, which had been punctured at its lower tip with an 18-gauge needle and placed inside a 1.5 mL Eppendorf tube. The tubes were spun at 2000 X *g* for 30 seconds and neutrophils were isolated as previously described [23]. After isolation, neutrophils were labeled with calcein-AM (5 μg/ml; Molecular Probes, Eugene, OR) for 30 minutes at 37°C, washed two times in PBS and resuspended at a concentration of 1 x 10^6^/mL.

Neutrophil chemotaxis was assessed using the Neuro Probe ChemoTx® Disposable Chemotaxis system (Neuro Probe Inc. Gaithersburg, MD). The wells of the 96-well plate were filled with various concentrations of KC. A polycarbonate filter (8 μm pores) with a hydrophobic ring around the area over each well was placed on the 96-well plate and calcein-labelled neutrophils were added to each ring. The chemotaxis chamber, consisting of the polycarbonate filter and 96-well plate, was incubated for 30 min at 37°C in 5% CO_2_ and then non-migrating neutrophils were removed from the upper side of the filter. The chemotaxis chamber was placed in a multi-well fluorescent plate reader (Synergy 4, BioTek, Winooski, VT) and the migrated cells were measured using the calcein fluorescence signal (excitation - 485 nm, emission - 530 nm). Neutrophil migration was expressed as a percent of the total number of neutrophils that were placed on the topside of the filter (% Total).

### Histology and immunohistochemistry

Lung sections were embedded in paraffin, cut into 4 μm sections, and stained with hematoxylin and eosin.

#### Double labeling for Terminal transferase dUTP nick end labeling (TUNEL) staining and cytokeratin

Lung tissue sections were deparaffinized, washed in xylene, rehydrated, permeabilized with proteinase K, and incubated with the TUNEL reaction mixture according to manufacturer instructions (*In Situ* Cell Death Detection kit, AP, Roche Applied Science, Indianapolis, IN). Negative controls were treated with labeling solution without terminal transferase. Immediately after TUNEL labeling the slides were washed three times in PBS, blocked with Protein Block (Dako, Carpinteria CA) and incubated for 2 hr in the dark at 37°C with the mouse monoclonal pan-cytokeratin antibody C11 (Abcam, Cambridge, UK) previously labeled with Alexa Fluor 555 (Invitrogen, Eugene, OR). Negative control slides were incubated with an isotype control mouse IgG1k labeled with Alexa Fluor 555 (BD Pharmingen, San Diego, CA). The slides were washed and immediately visualized with a fluorescent microscope.

#### Detection of anti-KC:KC immune complexes

Lung tissue sections from B6 and *lpr* mice exposed to MV + LPS were processed as previously described [21]. The sections were incubated with anti-KC antibody (Peprotech, Rocky Hill, NJ) followed by chicken anti-rabbit secondary antibody (Alexa 647, pseudocolor red) (Invitrogen), and then with anti-FcγRIII antibody (R&D Systems) followed by chicken anti-rat secondary antibody (Alexa 488, green), and finally with biotynylated anti-Ly-6 G antibody (eBioscience, San Diego, CA), used as a neutrophil marker, followed by streptavidin (Alexa 568, pseudocolor magenta). Lung tissues were counter-stained with Hoechst 33342 (Calbiochem, Gibbstown, NJ). The slides were evaluated using a PerkinElmer Ultra VIEW LCI confocal imaging system with Nikon TE2000-S fluorescence microscope using PlanApox20 objective and PlanApox60 or x100 immersion oil objective (numerical aperture [NA] 1.4) at room temperature. Ultra VIEW Imaging Suite software (version 5.5.0.4) was used for image processing.

### Statistical analysis

Statistical analysis was performed using two-factor ANOVA followed by the Bonferroni post-hoc analysis. One factor was “treatment” (SB or MV + LPS) and the other factor was “strain” (B6 or lpr). The analysis was designed to determine the overall effect of each of the factors, the presence of an interaction effect, and the comparison of “strain” for each level of “treatment”. Data was generated using GraphPad Prism. A *p* value of less than 0.05 was considered significant.

## Results

### The neutrophil response to MV + LPS was attenuated in the *lpr* mice

**Figure 1:**
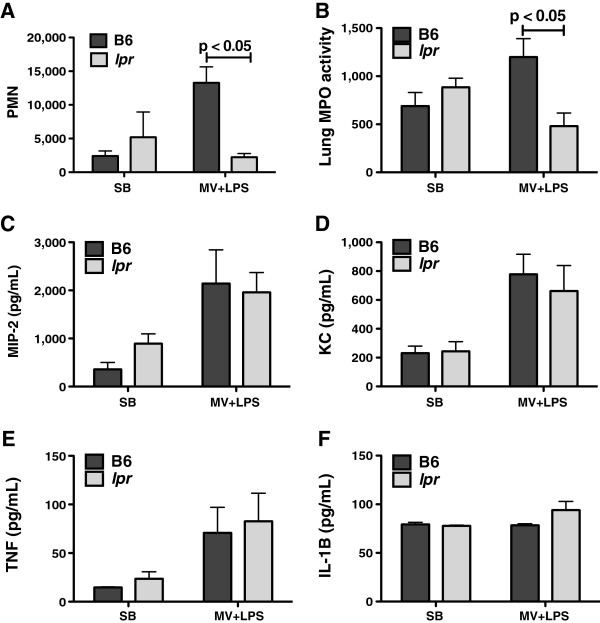
**Inflammatory response.** C57BL/6 (B6) and Fas-deficient *lpr* mice received intratracheal installations of either PBS followed by four hours of spontaneous breathing (SB), or *E. coli* LPS, 15 ng/kg, followed by 4 hrs of mechanical ventilation (MV) with tidal volumes of 10 mL per kilogram, FiO_2_ of 0.21, PEEP = 3 and respiratory rate = 150 breaths per minute. In response to the combination of MV and LPS, the B6 mice showed significantly more total neutrophils (PMN) in the BAL fluid than the lpr mice (**A**). A similar pattern was seen for the lung MPO activity, which is a measure of the total neutrophil content in the lung (**B**). MV + LPS was associated with increases in the lung homogenate concentrations of the cytokinesTNF-α, MIP-2, and KC, and these similar in the B6 and the *lpr* mice; MV + LPS had no effect on IL-1β (**C**-**F**). n = at least 6/group.

There were significantly less PMN in the BAL of *lpr* mice exposed to LPS + MV than in the B6 mice (2.2 x 0.5 x 10^3^ vs 1.3 ± 0.2 x 10^4^ cells, p < 0.05)(Figure 1-A). A similar pattern was seen for lung MPO activity (Figure 1-B). However, the lung concentrations of the PMN chemoattractants KC and MIP2 (CXCL2), although increased in response to MV + LPS, were similar in the *lpr* and B6 mice; this was also true for the pro-inflammatory cytokine TNFα (Figure 1, C-F). Interleukin 6, GM-CSF, and VEGF were not affected by the administration of MV + LPS and were similar in the B6 and *lpr* mice (data not shown). These data suggest that both lung PMN recruitment and airspace PMN migration were impaired in the absence of functional Fas, but the difference in neutrophil recruitment seen in this model cannot be explained by differences in KC or MIP-2 release.

### Other parameters of lung injury were similar in B6 and *lpr* mice

#### Permeability response

**Figure 2:**
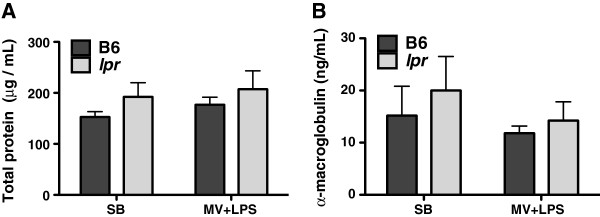
**Permeability response.** Total BAL protein (**A**) and concentrations of the large molecular weight protein alpha macroglobulin (**B**) in the BAL fluid of C57BL/6 (B6) or Fas-deficient *lpr* mice that received intratracheal installations of either PBS or LPS, followed by either spontaneous breathing (SB) or four hours of mechanical ventilation (MV). There were no significant differences among any of the groups studied. n = at least 6/group.

We and others have postulated that the Fas/FasL system leads to lung injury by inducing apoptosis of pneumocytes, resulting in disruption of the alveolar-epithelial barrier and non-cardiogenic pulmonary edema. However, in the present study the BAL concentration of total protein and of the serum protein α-macroglobulin were similar in the *lpr* and B6 mice, despite the difference in PMN numbers (Figure 2). The lack of difference may be due to a low level of injury, as supported by the lack of overall change in the MV + LPS group as compared with the SB group; indeed the model was designed so that the ventilatory pattern and the LPS dose would cause minimal or no injury by themselves, so as to determine the very first components of the injury response. The data suggest that neutrophil changes precede permeability changes in ventilated mice exposed to LPS, and that the role of the Fas/FasL system on neutrophil recruitment precedes its disruptive effect on the alveolar-capillary barrier.

#### Apoptotic response

**Figure 3:**
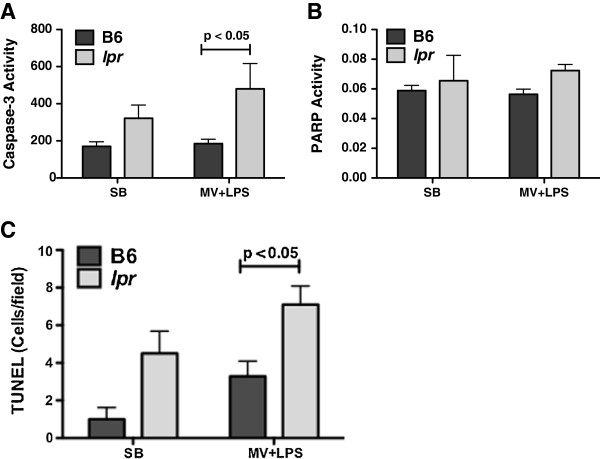
**Apoptotic response.** Caspase-3 activity (**A**) and Poly ADP ribose polymerase (PARP) (**B**) in the lungs of C57BL/6 (B6) or Fas-deficient LPR mice treated with intratracheal installations of either PBS or E. coli LPS, 15 ng/kg, followed by either spontaneous breathing (SP) or four hours of mechanical ventilation (MV) with tidal volumes of 10 mL per kilogram. Caspase-3 activity was significantly higher in the lpr mice exposed to MV + LPS. n = at least 6/group. Double-labeling for TUNEL (green) and cytokeratin (red) reveals that the TUNEL positive cells are located in the alveolar wall, but most of them are cytokeratin negative.

Interestingly, the activity of caspase-3, often used as a surrogate for apoptosis, was increased in the spontaneously breathing *lpr* mice, and this increase reached significance in the MV + LPS group (185 ± 24 vs 480 ± 137, p < 0.05)(Figure 3-A). PARP, a downstream target of active caspase-3, showed similar activity in the *lpr* and B6 mice (Figure 3-B). Double labeling of tissue sections for TUNEL and cytokeratin revealed that in both the *lpr* and the B6 mice, the apoptotic cells were located primarily in the alveolar walls, but were cytokeratin-negative (Figure 3-C). Thus, contrary to our expectations, there was increased caspase-3 activity in the *lpr* mice exposed to MV + LPS, and the apoptotic cells were mostly cytokeratin-negative cells localized to the alveolar walls.

#### Tissue response

**Figure 4:**
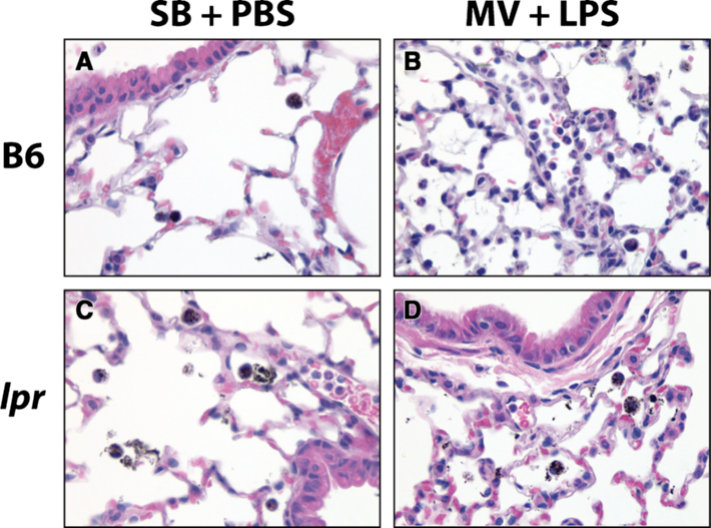
**Tissue respons.** C57BL/6 or Fas-deficient lpr mice were exposed to either intratracheal installations of PBS followed by spontaneous breathing or to intratracheal installation of LPS, 15 ng/kg, followed by four hours of mechanical ventilation (MV) with tidal volumes of 10 mL per kilogram. Lung tissue sections were stained with hematoxylin and eosin to demonstrate the pattern of injury. The instillate contained 2.5% colloidal carbon to identify the instilled areas. Spontaneously breathing mice instilled with PBS showed normal lung architecture, regardless of strain (**A**, **C**). The B6 mice exposed to MV and LPS showed intra-alveolar and interstitial neutrophilic infiltrates, as well as thickening of the alveolar walls and occasional fibrin deposition (**B**).

The lungs of spontaneously breathing B6 and *lpr* mice showed normal architecture (Figure 4). The lungs of B6 mice exposed to MV + LPS showed thickening of the alveolar walls, intra-alveolar neutrophilic infiltrates, and intra-alveolar fibrin strand deposition. In contrast, the lungs of *lpr* mice exposed to MV + LPS retained their normal architecture. The LPS was administered with inert colloidal carbon, which on the tissue sections was taken up by macrophages and appears as black granulate material in the cytoplasm. This indicates that the sections depicted were all exposed to instillate -an important point as intratracheal instillations are patchy and normal tissue can simply reflect a non-instilled area.

#### Systemic response

**Figure 5:**
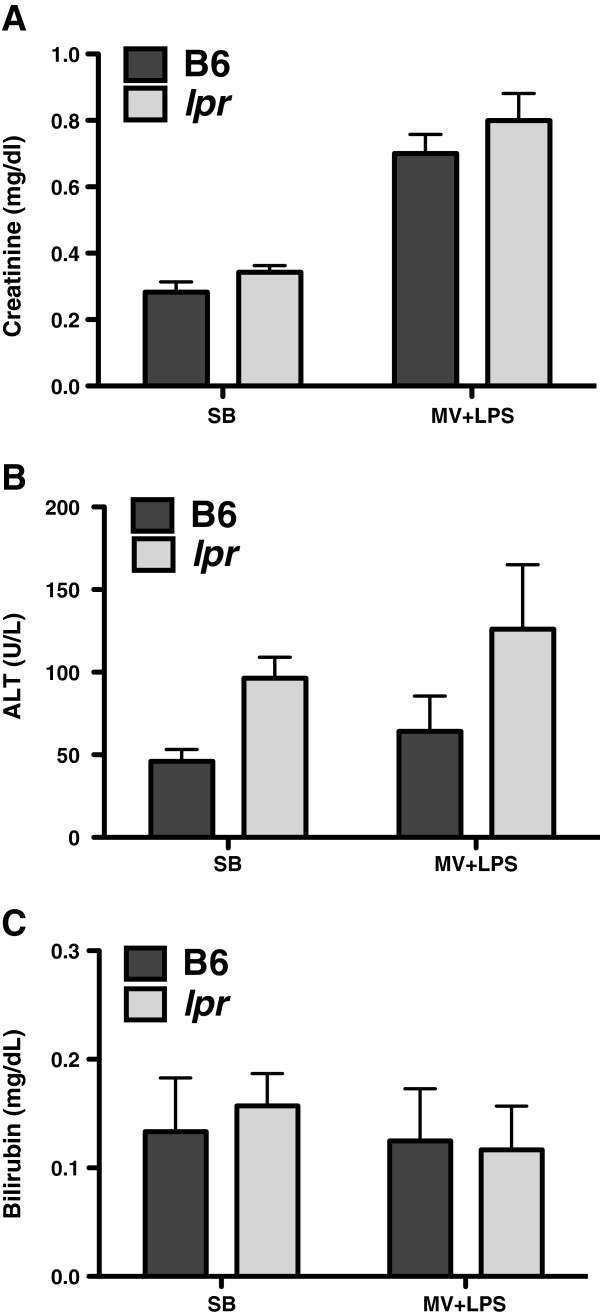
**Distal Organ Injury.** Creatinine (**A**), alanine aminotransferase (ALT) (**B**) and total bilirubin (**C**) in the serum of C57BL/6 (B6) or Fas-deficient *lpr* mice exposed to either intratracheal instillations of PBS followed by spontaneous breathing (SB), or intratracheal instillations of LPS, 15 ng/kg, followed by 4 hr of mechanical ventilation (MV) with Tv = 10 cc/kg, FiO_2_ = 0.21% and PEEP = 3. At the times tested, the combination of MV + LPS resulted in an increase in creatinine, but not ALT or bilirrubin. There was no difference in the creatinine concentrations of the B6 or *lpr* mice. n = at least 6/group.

We have previously reported that the combination of MV + LPS for 6 hours (2 hr longer than the present study) is associated with distal organ injury, in particular biochemical and histologic evidence of kidney and liver damage [24]. In this study, we also noticed an increase in serum creatinine in the mice exposed to MV + LPS, but this increase was strain-independent (Figure 5-A). Interestingly, the *lpr* mice had increased serum AST concentrations at baseline, and this was not further increased by the addition of MV + LPS (Figure 5-B). Total bilirubin was similar in *lpr* and B6 mice, and was not affected by MV + LPS (Figure 5-C). Thus, at this early time the evidence of distal organ injury was limited to increased creatinine, and this increase was not dependent on the Fas/FasL system.

### The differences in BAL neutrophils are not explained by differences in B6 and *lpr* neutrophil chemotaxis

**Figure 6:**
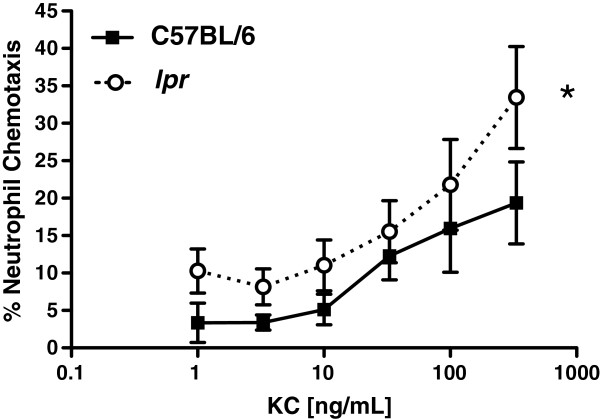
**Neutrophil chemotaxis.** Chemotaxis towards KC of peripheral blood neutrophils isolated from *lpr* and B6 mice. Note the increased response of neutrophils from *lpr* mice at the higher doses of KC. * = p < 0.05, n = 3.

An impairment in *lpr* neutrophil chemotaxis towards KC could explain the attenuation of BAL neutrophils seen in the *lpr* mice exposed to MV + LPS. Therefore, we compared the chemotactic ability of neutrophils isolated from B6 and *lpr* mice to KC *in vitro.* However, we noticed no impairment of chemotaxis in the *lpr* neutrophils; if anything, chemotaxis was slightly increased in the *lpr* neutrophils as compared with the B6 neutrophils (Figure 6).

### Anti-KC autoantibody:KC immune complex deposition

**Figure 7:**
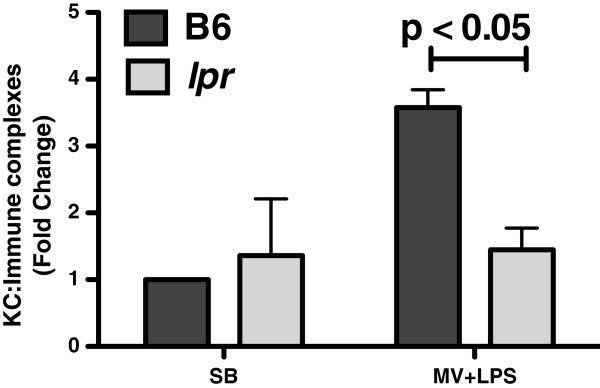
**Soluble KC:antiKC immune complexes in BAL fluid.** Soluble anti-KC:KC immune complexes as measured by ELISA in the BAL fluid from B6 and *lpr* mice allowed to breath spontaneously, or treated with *E. coli* LPS followed by 4 hours of mechanical ventilation with Tv = 10 cc/kg, FiO_2_ = 0.21, PEEP = 3. Note that the B6 mice had a significant fold increase in immune complex concentrations as compared with the *lpr* mice.

**Figure 8:**
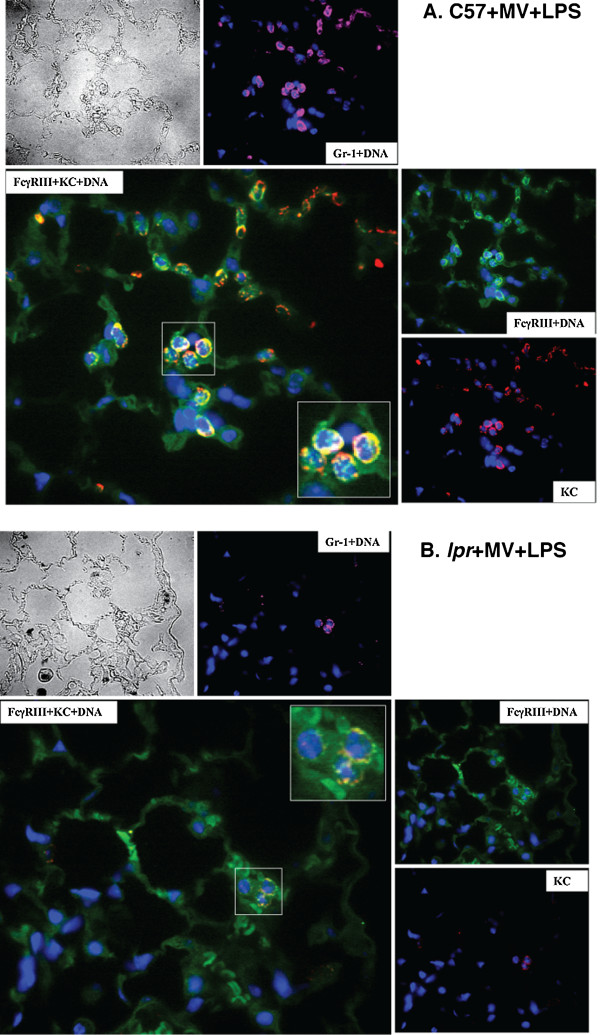
**Anti-KC autoantibody:KC complex deposition in the lungs.** FcγRIII (green) and KC (red) in tissue sections from B6 (**A**) and lpr (**B**) mice treated with intratracheal E. coli LPS, 15 ng/kg, and exposed to 4 hours of mechanical ventilation (MV) at Tv = 10 cc/kg, FiO2 = 0.21, PEEP = 3. FcγRIII is shown in green, KC is shown in red, and Gr1 (staining leukocytes) is shown in pink. Nuclei are shown in blue. There is an increase signal for both KC and FcγRIII in the B6 mice as compared with the lpr mice. Note the colocalization of KC and FcγRIII.

A new line of research suggests that mammals form autoantibodies against chemokines such as IL8 in humans and KC in mice, and immune complexes derived from these autoantibodies can induce inflammation by binding to Fcγ receptors on leukocytes, endothelial cells and other cells. Measurement of anti-KC:KC complexes in the BALF revealed an increase in B6 mice treated with MV + LPS, which was not seen in the *lpr* mice (Figure 7) We also used immunofluorescence staining and confocal microscopy to investigate if there were differences in the deposition of anti-KC:KC complexes in ventilated B6 and *lpr* mice. The alveolar walls of the ventilated B6 mice contained abundant leukocytes, identified based on Gr-1 positivity (Figure 8). The leukocytes expressed the receptor FcγRIII on their membrane surface. KC co-localized with the FcγRIII receptors. In contrast, there were, in general, less Gr-1 positive cells in the lungs of the lpr mice, and there was also decreased deposition of KC in the tissue. Thus, the formation of anti-KC:KC IC was attenuated in *lpr* mice, and this was associated with decreased KC deposition in the tissues.

**Figure 9:**
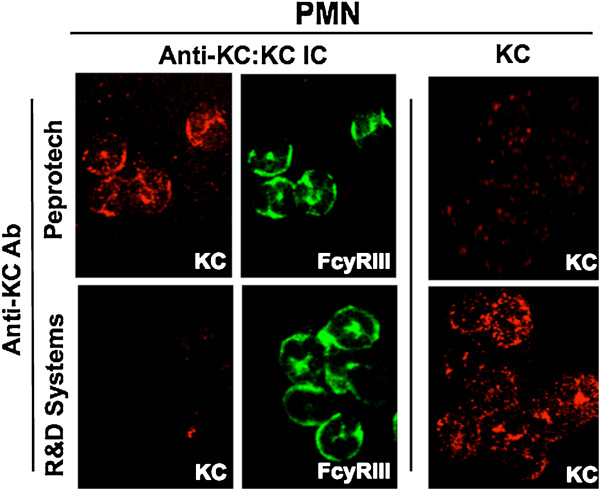
**The detection of anti-KC:KC immune complex is specific.** Mouse PMN were purified and incubated with either anti-KC-KC immune complexes (first two columns) or with KC alone (third column). Note that the Peprotech antibody, detected only anti-KC-KC immune complexes, whereas the R&D antibody detected only KC. The FcγRIII was labeled green and KC was labeled red.

To confirm the specificity of the anti-KC:KC labeling, mouse PMN were purified and incubated with either anti-KC-KC immune complexes (Figure 9, first two columns) or with KC alone (Figure 9, third column). Then, the cells were labelled with either the Peprotech antibody, which detected the immune complexes, or an R&D antibody, which detected only KC.

**Figure 10:**
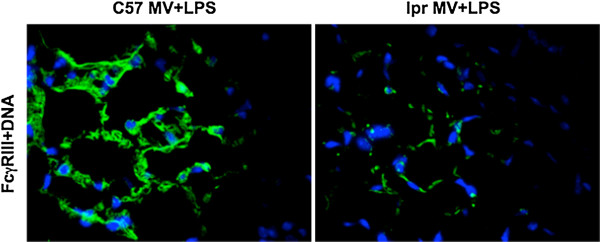
**Expression of FcγRIII is lower in lpr mice.** C57BL/6 (B6) and Fas-deficient lpr mice received intratracheal installations of E. coli LPS, 15 ng/kg, followed by 4 hrs of mechanical ventilation (MV) with tidal volumes of 10 mL per kilogram, FiO2 of 0.21, PEEP = 3 and respiratory rate = 150 breaths per minute. Note decreased expression of FcγRIII (green) in the lpr mice. Nuclei are labeled blue.

Finally, we tested expression of FcγRIII in WT and *lpr* mice treated with MV + LPS, and found it to be lower in the *lpr* mice (Figure 10).

## Discussion

The goal of this study was to determine whether Fas deficiency is protective in mechanically ventilated mice exposed to intratracheal LPS. The main finding was that Fas-deficient *lpr* mice showed decreased neutrophil recruitment in response to combined mechanical ventilation and LPS, even though the cytokine, permeability, and apoptotic responses were similar to those of B6 mice. Interestingly, despite the presence of similar concentrations of KC in the BAL of *lpr* and B6 mice, only the B6 mice showed extensive KC deposition in the lungs, and this was associated with the presence of anti-KC:KC complexes in the BALF and lung tissue.

In this study, we focused on the initial events of the injury response by studying mice four hours after the initiation of injury, which was induced by combining a very low dose of endotoxin with mechanical ventilation. The main finding was that a functional Fas/FasL system was required for neutrophil migration into the airspaces of the lungs of mechanically ventilated mice exposed to LPS. The changes in BAL neutrophils seen in this study were associated with changes in lung myeloperoxidase (MPO) activity, and because lung MPO activity is a measurement of the total content of neutrophils in the lungs, the data suggests that the Fas/FasL system is required for both neutrophil migration into the airspaces and for neutrophil recruitment into the lungs. This observation confirms other studies that suggest a key role for the Fas/FasL system in neutrophil migration into the airspaces; for example, activation of Fas in the lungs of mice and rabbits results in a neutrophilic alveolitis (1, 2), whereas pharmacologic blockade of the Fas/FasL system attenuates the neutrophilic response to bacteria and bacterial products (3-6).

One potential explanation for the decreased migration of neutrophils into the airspaces of the Fas-deficient mice is that Fas ligation induces release of neutrophilic cytokines such as KC in macrophages and in lung epithelial cells *in vitro* (7-9). Thus, we had expected to see lower concentrations of KC in the mechanically ventilated *lpr* mice exposed to LPS, as compared with the B6 animals. However, this was not the case, and the difference in neutrophil migration was not due to differences in soluble KC concentrations.

Another potential mechanism that would explain differences in neutrophil migration with similar concentrations of soluble KC is a difference in neutrophil chemotaxis of *lpr* and B6 neutrophils. However, our chemotaxis studied showed that, if anything, neutrophils from *lpr* mice have slightly increased chemotaxis to KC, strongly suggesting that differences in chemotaxis are not the reason for the attenuation in the neutrophilic response seen in the lpr mice.

Neutrophil recruitment and migration appears to be partly dependent on the formation of anti-KC:KC immune complexes, which can bind Fcγ receptors in local leukocytes, enhancing the inflammatory response [22]. For example, the generation of anti-KC:KC immune complexes in the lungs of mice is followed by acute inflammatory lung injury, and this injury requires the presence of Fcγ receptors [21]. Furthermore, mice lacking γ chains show attenuated injury in response to LPS, suggesting that this process is relevant for inflammation secondary to bacterial products [21]. The human equivalent of anti-KC:KC complexes are anti-IL8:IL8 complexes, and these are present in the lungs of patients with ARDS [25,26]. In our study, we found that there are less anti-KC:KC complexes in the lungs of the *lpr* mice, even though the concentrations of KC were similar to those in the B6 mice. Thus, it is possible that the lower numbers of neutrophils seen in the *lpr* mice were due to decreased formation and deposition of anti-KC:KC complexes in the lungs. Additional studies are necessary to confirm this hypothesis, and it remains possible that differences in other unmeasured chemotactic agents account for the differences in neutrophil recruitment. The mechanism linking the Fas/FasL system with impaired formation and deposition of immune complexes remains unclear. To date, *lpr* mice, particularly the MRL/*lpr* strain are known to generate autoantibodies that can result in a lupus-like syndrome [27].

A surprising finding in the present study was that there was no increase in the markers of permeability or apoptosis in the B6 mice exposed to mechanical ventilation and LPS, as compared to the *lpr* mice; instead, the injury response was limited to neutrophilic inflammation and cytokine release. One explanation is that neutrophil recruitment precedes the development of tissue injury in our model, in which the mice were studied relatively early, four hours after the onset of ventilation. Less clear is the finding that the *lpr* mice actually had increased caspase-3 activity and TUNEL positive cells compared with the B6 mice. We do not have a clear explanation for this finding, but they seem to be specific to the LPS + MV model, because in another model using mechanical ventilation in which WT and lpr mice were exposed to pneumonia virus of mice (PVM) prior to four hours of MV, we found a decrease in caspase-3 activity in the *lpr* mice [28].

It is important to emphasize that the decrease in neutrophils seen in the *lpr* mice does not imply “protection”. It is unclear whether the lung neutrophilic response is directly associated with negative outcomes in ALI/ARDS or not. Most studies of lung injury presume that neutrophilia or increased concentrations of cytokine are deleterious, but all of these studies are limited in that they do not effectively reproduce the series of events seen in the clinical setting, where patients with multiple comorbidities are intubated for prolonged periods of time. Our study should be interpreted as knowledge on the mechanisms of that initiate lung injury, but not extrapolated to the ultimate results of that injury.

## Conclusions

In summary, we report that in B6 mice, the combination of mechanical ventilation and LPS is associated with recruitment of neutrophils to the lungs and with deposition of anti-KC:KC immune complexes. In comparison, mice deficient in Fas recruited lower numbers of neutrophils, and this was associated with less deposition of immune complexes in the lungs. We conclude that a functioning Fas/FasL system is required for a full neutrophilic response to LPS in mechanically ventilated mice.

## Competing interests

The authors declare no financial, consulting or personal relationships with other people or organizations that could influence this work.

## Authors’ contributions

SG performed the animal experiments; AWF performed the histology and contributed to the redaction and editing of the manuscript; WAA contributed in the development of the animal model; SEG performed the chemotaxis assays and contributed with the editing of the manuscript; AK performed the immune complex measurements, including immunohistochemistry, and participated in the writing of the manuscript; JMF assisted AK with revising the manuscript; GMB conceived and directed the experiments and wrote the manuscript. All authors read and approved the final manuscript.
